# P-1904. The Interaction of Serum Biomarkers and Lymphopenia on Pulmonary COVID-19 Illness Progression in Hospitalized Patients

**DOI:** 10.1093/ofid/ofae631.2065

**Published:** 2025-01-29

**Authors:** Gregorio Benitez, Matthew Kaczynski, Stephanos Vassilopoulos, Athanasios Vassilopoulos, Fadi Shehadeh, Karen T Tashima, Eleftherios Mylonakis

**Affiliations:** University of Michigan, Ann Arbor, Michigan; The Warren Alpert Medical School of Brown University, Providence, Rhode Island; Warren Alpert Medical School of Brown University, Providence, Rhode Island; Division of Internal Medicine, Warren Alpert Medical School of Brown University, Rhode Island Hospital, Providence, Rhode Island, Providence, Rhode Island; Houston Methodist Research Institute, Houston, TX, Houston, Texas; Warren Alpert Medical School of Brown University, Providence, Rhode Island; Houston Methodist Hospital, Houston, TX, Houston, Texas

## Abstract

**Background:**

Low absolute lymphocyte count (ALC) as well as elevated levels of plasma nucleocapsid antigen (N-antigen) and C-reactive protein (CRP) are independently associated with COVID-19 clinical outcomes. We evaluated the potential additive interaction of these three prognostic markers on the progression of pulmonary COVID-19 illness.

**Figure:**
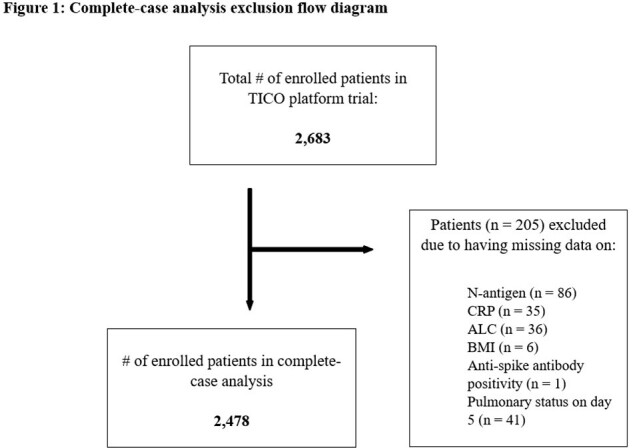

**Methods:**

We utilized publicly available data to perform a secondary analysis of hospitalized adults who enrolled into the Therapeutics for Inpatients with COVID-19 platform trial (NCT04501978). Plasma N-antigen level > 1000 ng/L, CRP level > 75 mg/L, and ALC < 1.5x10^9^/L, all measured at baseline, were the three prognostic markers of interest. The outcome of interest was worsened pulmonary status, dichotomized as either worse or same/better than baseline status on a 7-point ordinal scale, at day 5. Estimated coefficients from modified multivariable Poisson regression were utilized to calculate risk ratios. We then calculated relative excess risk due to interaction (RERI) to evaluate departure from additivity. We present a measure of excess risk due to the respective two-way interactions when one of the markers is absent (RERI2), a measure of excess risk due to the three-way interaction (RERI3), and a total summation of excess risk (TotRERI).

**Figure:**
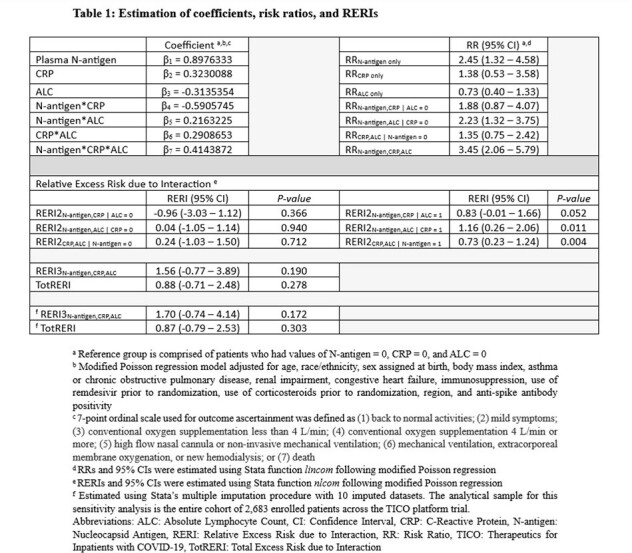

**Results:**

A total of 2,478 enrolled patients were included in our complete-case analysis (Figure 1). The interaction of low ALC, elevated plasma N-antigen level and elevated CRP level on worsened pulmonary status at day 5 may be super-additive (TotRERI = 0.88, 95% confidence interval (CI): -0.71 – 2.48) (Table 1). This 88% excess risk of worsened pulmonary status is mainly due to the explicit three-way interaction (RERI3: 1.56, 95% CI: -0.77 – 3.89). Sensitivity analysis utilizing multiple imputation to address missing data revealed similar estimates: TotRERI: 0.87 (95% CI: -0.79 – 2.53) and RERI3: 1.70 (-0.74 – 4.14).

**Figure:**
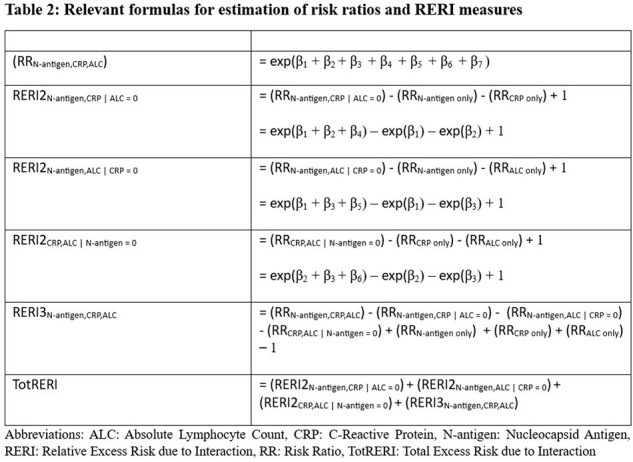

**Conclusion:**

Our data suggest that the interaction of low ALC, elevated plasma N-antigen level and elevated CRP level may be associated with excess risk of worsened pulmonary COVID-19 illness among hospitalized patients. Further evaluation regarding the interaction of prognostic markers in identifying patients at high-risk of pulmonary deterioration is supported.

**Disclosures:**

Eleftherios Mylonakis, MD, PhD, BARDA: Grant/Research Support|Basilea: Advisor/Consultant|Chemic Labs/KODA Therapeutics: Grant/Research Support|Cidara: Grant/Research Support|Leidos Biomedical Research Inc./NCI: Grant/Research Support|NIH SciClone Pharmaceuticals: Grant/Research Support|NIH/NIAID: Grant/Research Support|NIH/NIGMS: Grant/Research Support|Pfizer: Grant/Research Support|Regeneron Pharmaceuticals, Inc.: Grant/Research Support

